# Supplementary material for: Determining the Preferred Orientation of Silver-Plating via X-ray Diffraction Profile
Source: Nanomaterials (Basel). 2021 Sep 17;11(9):2417. doi: 10.3390/nano11092417 (PMC8466463; doi:10.3390/nano11092417)
Supplement: Supplementary file 1 [file nanomaterials-11-02417-s001.zip › nanomaterials-1360596-supplementary.pdf]

nanomaterials

Determining the Preferred Orientation of Silver-Plating via X-ray Diffraction Profile

Taotao Li [1,*], Liuwei Zheng [2], Wanggang Zhang [2,*] and Pengfei Zhu [3]

[1] School of Mechanical Engineering, North University of China, Taiyuan 030051, China
[2] College of Materials Science and Engineering, Taiyuan University of Technology, Taiyuan 030024, China; zhengliuwei@tyut.edu.cn
[3] College of Mechanical and Electrical Engineering, Central South University, Changsha 030051, China; zhupengfei4105@163.com
* Correspondence: litaotao12138@nuc.edu.cn (L.T.); zhangwanggang@tyut.edu.cn (Z.W.)

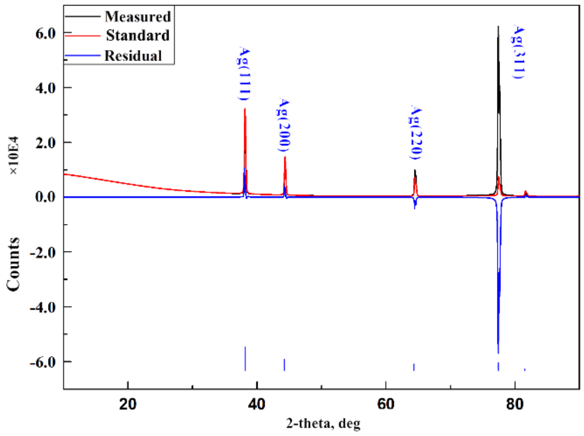

Figure S1. The agreement between the measured and theoretical XRD profile for the silver film with no preferred orientation correction.

Since the silver film is textured, it was not possible to obtain a good fit and there are positive and negative deviations intensity, which are illustrated in the lower curve of Figure S1. These residual deviations are due to the texture and contain information about the orientation distribution.

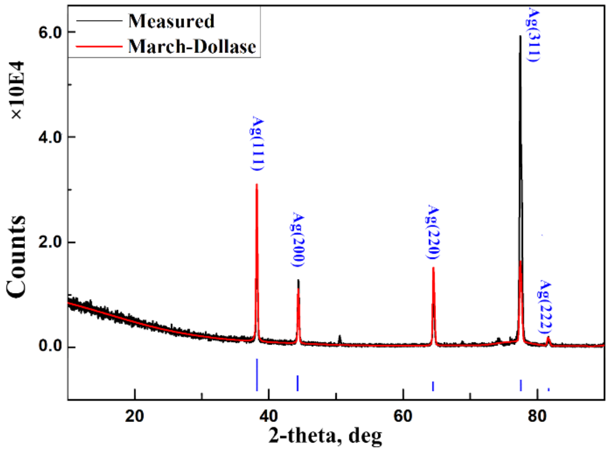

Figure S2. The agreement between the measured and calculated XRD profile for the silver film with March-Dollase correction for preferred orientation.

Table S1. Refined crystal structure, microstructure, and preferred orientation index for silver film in materials analysis using diffraction (MAUD).

Refined parameters		Microstructure Line Broadening model Size-strain model	Delft Popa rules	Model	Preferred orientation Exponential Harmonic Index
		anisocryst size 0	1552.4		
		anisocryst size 1	999.6	$C_4^{1,1}$	0.609
R_{wp}	**10.3%**	anisocryst size 2	471.0		
		anisocryst size 3	316.8		
		anisocryst size 4	89.2	$C_6^{1,1}$	0.278
		anisocryst size 5	-1811.9		
		anisocryst size 6	1567.2		
constant	4.089671	aniso microstrain 0	2.56E-4	$C_8^{1,1}$	-0.970
		aniso microstrain 1	1.11E-3		

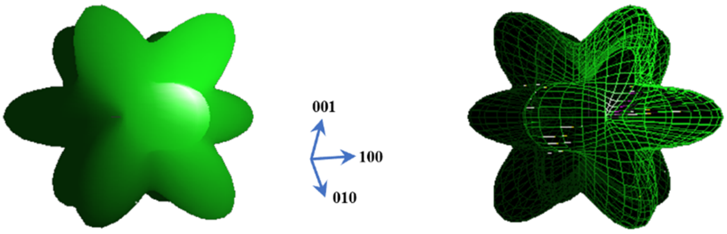

Figure S3. The simulated solid and wireframe crystallite with Popa rules.

Yet, it is an indispensable procedure to perform the quantitative microstructure analysis before texture analysis in Rietveld analysis. In the microstructure correction, 8 anisocrystallite parameters of the averaged silver crystallite and two anisomicrostrain parameters are refined with the Popa rules with the simulated solid and wireframe crystallites as shown in Figure 1c [1-5]. All the Popa parameters correspond exactly to those reported in table S1 and the crystallite value corresponds to the diameter of the crystallite (the sing of the parameter is conserved) as shown in Figure S3. The extended feeler in the simulated crystallites is accounted for the increase of the {311} plane, resulting in the increased {311} peak in the measured XRD profile. With the finish of the quantitative microstructure analysis, then the texture analysis can be performed in the last place.

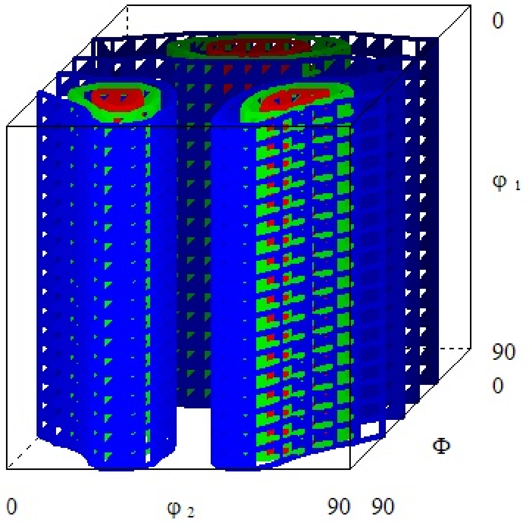

Figure S4. the constructed three-dimensional orientation distribution function constructed with the classical pole figure analysis.

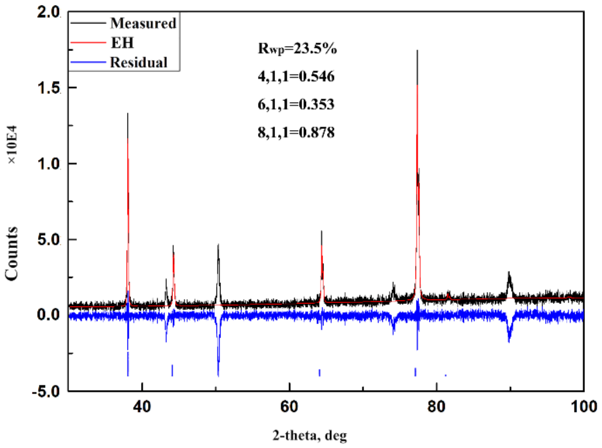

Figure S5. the agreement between measured and calculated XRD profile for the silver film (0.3A/dm^2, 10min) following Rietveld refinement with exponential harmonics description (EH) for preferred orientation correction.

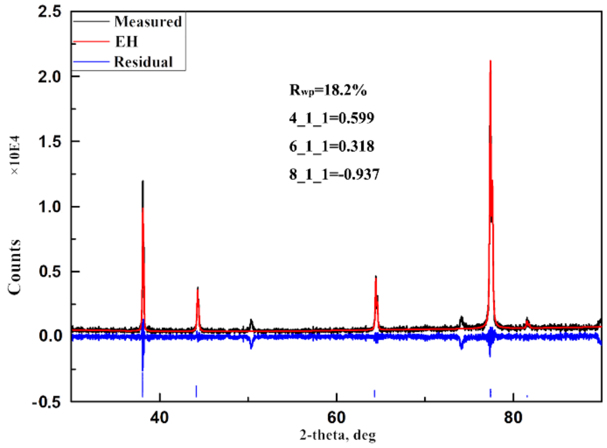

Figure S6. the agreement between measured and calculated XRD profile for the silver film (0.3A/dm^2, 20min) following Rietveld refinement with exponential harmonics description (EH) for preferred orientation correction.

Table S2. Refined preferred orientation index for silver film in the current density (0.3A/dm^2) and different plating times with materials using diffraction (MAUD).

samples		Exponential Harmonic Index	
**Current (A/dm^2)**	**Time (min)**		
0.3	10	$C_4^{1,1}$	0.546
		$C_6^{1,1}$	0.353
		$C_8^{1,1}$	-0.878
0.3	20	$C_4^{1,1}$	0.599
		$C_6^{1,1}$	0.318
		$C_8^{1,1}$	-0.973
0.3	40	$C_4^{1,1}$	0.609
		$C_6^{1,1}$	0.278
		$C_8^{1,1}$	-0.970

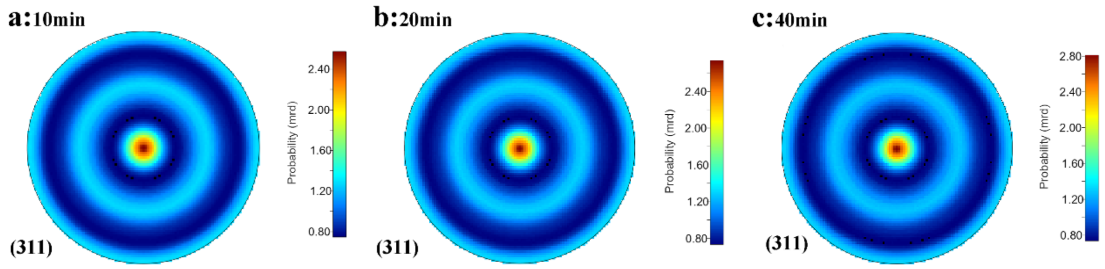

Figure S7. The constructed 2D 311 pole figure with the index of the exponential harmonic in Table S2, a: 10min, b: 20min, c: 40min.

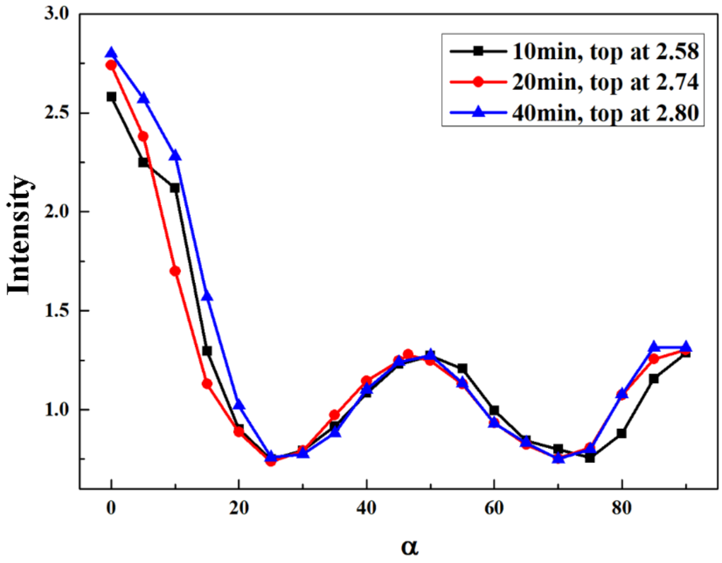

Figure S8. Pole-axis distribution plots for (311) silver film at different plating times derived from the Rietveld method and quantitative texture analysis (RM+QTA).

With the Rietveld method and quantitively texture analysis, the preferred orientation of the silver films at the current density of 0.3A/dm^2 in different plating times (10, and 20 min) are defined as shown in Figure S5 and S6. Then, the corresponding exponential harmonics index is listed in Table S2 and can be used for direct comparison. The pole figures with different plating times are constructed with Maud as shown in Figure S7. It can be confirmed by the pole-axis distribution plots for (311) that the preferred orientation index $C_4^{1,1}$ increase with the increase of the plating time.

[1] N.C. Popa, D. Balzar, Journal of Applied Crystallography 35 (2002) 338.
[2] Popa N C, Teodorescu C M, Frunza S Journal of Applied Crystallography, 43 (2010) 5.
[3] Popa N C, Balzar D, Journal of Applied Crystallography, 41 (2008), 615.
[4] Popa N C, Journal of Applied Crystallography, 25 (1992), 611.
[5] Popa N C. Journal of Applied Crystallography, 31 (2010), 176.
